# Different understandings, different responses: experiences of racism among highly educated, second generation Black Germans

**DOI:** 10.3389/fsoc.2025.1450981

**Published:** 2025-02-25

**Authors:** Eunike Piwoni

**Affiliations:** Faculty of Social and Educational Sciences, University of Passau, Passau, Germany

**Keywords:** qualitative interview, Black Germans, racism, anti-racism, Germany

## Abstract

This article argues that there is a close relationship between individuals’ understandings of specific incidents of racism, their ideas of how racism operates, and their (repertoires of) responses to such incidents. The argument is based on a qualitative interview study with 21 highly educated Black Germans with at least one parent born outside Germany, and draws on both the extant literature on responses to experiences of ethnoracial exclusion and research into how people make sense of such experiences. The analysis specifically explores two contrasting types of interviewees: Type 1 felt that they were constantly and potentially always affected by racism and had a broad knowledge of racism. These interviewees recounted many different incidents, many of which they clearly labelled as “racist.” Type 1 interviewees reported a variety of response options, with direct confrontation being one of them. In stark contrast, Type 2 respondents tended to normalise the relatively few incidents they mentioned or indicate only feelings of unease. They also believed that they were largely unaffected by racism, had a less deep understanding of racism and tended to respond to incidents of exclusion in ways that allowed the encounter to continue without disruption. Overall, the study calls for greater attention to racialised people’s meaning-making in relation to concrete incidents of exclusion and to their knowledge of racism. This requires methodological adaptations to qualitative interview research, which remains the most popular method for exploring experiences of racism. In particular, the study highlights the importance of understanding the ways in which respondents talk about their experiences (categorisation, indication of feelings of unease, and normalisation). It also emphasises the need to go beyond considering only interviewees’ responses to direct questions about their experiences of racism and/or discrimination and/or incidents clearly categorised by interviewees as, for example, “racist.” Moreover, reconstructing interviewees’ knowledge about racism offers a path towards understanding not only their sense-making but also their repertoires of responses. This, in turn, provides insight into why individuals of comparable class position and educational background respond to racism in different ways.

## Introduction

In ethnic and migration studies, research on people’s responses to stigmatisation, discrimination, ethnoracial exclusion and racism in different contexts is ever-growing (see, for example, Bickerstaff, 2012; Lamont et al., 2016; Witte, 2018; Ellefsen and Sandberg, 2022; Ellefsen et al., 2022; Yurdakul and Altay, 2023; Dazey, 2023; Drouhot, 2023).^1^ These studies document an abundance of responses including, for instance, ignoring an offensive comment, confronting someone with their racist behaviour, being polite (as a strategy to fight being stereotyped), or the emphasising of one’s class status to name just a few (for an overview and a systematisation of responses see Piwoni, 2023).

Alongside this growing knowledge about the variety of strategies and responses to incidents of ethnoracial exclusion, the question of *why* racialised individuals (choose to) respond to such experiences in different ways has come to the fore. In their comparative study on African Americans in the US, Black Brazilians in Brazil and Mizrahim, Arab Palestinians, and Ethiopian Jews in Israel Lamont et al. (2016) seek to explain why these groups respond differently to experiences of exclusion and do so by pointing to macro-level factors such as cultural repertoires available within a specific national context. In the US, for example, there is widespread cultural knowledge about how to recognise racism. This knowledge is not least a consequence of the Civil Rights Movement and makes African Americans more confident than Black Brazilians in responding to racist incidents with confrontation.

Focusing on in-group differences in how people respond to discrimination, Doering and Peker’s (2022) study of Muslims in Quebec shows that experiences and responses within the same ethnoreligious minority can differ in a given sociopolitical context, not least because of variety in whether individuals interpret secularist restrictions as discrimination. Thus, there is growing evidence that people’s understandings of racism and discrimination, their ideas on where and how racism and discrimination occur, and their sense-making of potentially exclusionary incidents correlate with their repertoires of antiracist responses and strategies (see also Bickerstaff, 2012, p. 124 for a similar remark).

The present study aims to focus explicitly on this relationship between understandings of racism and racist incidents on the one hand and responses to such incidents on the other in terms of in-group differences, and to explore these relationships and responses in detail through the analysis of in-depth interviews with 21 Black Germans conducted between 2018 and 2021. Conceptually, the study builds on the author’s previous research with interviewees from different migrant backgrounds (including the Black German interviewees in this study), which has focused on how individuals recognise and make sense of experiences and incidents of ethnoracial exclusion (Piwoni, 2024a).^2^ This research has shown that racialised individuals do not always “comprehend” incidents as “racist,” but may often only “sense” racism and indicate feelings of unease without explicitly categorising specific experiences as “racist.” Alternatively, they may normalise incidents of exclusion. The present study builds on these findings and demonstrates, firstly, that these different ways of understanding experiences of exclusion are associated with different types of responses and, secondly, that there are in-group differences in how interviewees make sense of and respond to their experiences. More specifically, the study focuses on and contrasts two types of interviewees: Type 1 felt that they were constantly and potentially always affected by racism. Interviewees of this type recounted many different incidents many of which they clearly understood as “racist.” For Type 1 interviewees, directly addressing and confronting racism was an option. In contrast, Type 2 respondents tended to normalise the relatively few incidents they mentioned, or to indicate only feelings of unease. They also believed that they personally were largely unaffected by racism and they tended to respond to incidents of exclusion in a non-confrontational way.

The remainder of the article is structured as follows: First, I provide an overview of two strands of literature: the literature that documents and discusses responses to ethnoracial exclusion in various contexts, and the literature on individuals’ perceptions of racism and incidents of exclusion. Second, I contextualise the present study by introducing Germany as a case and providing background information on Black people in Germany. Third, I outline my methods of data collection and analysis before presenting the findings of the study. In the conclusion, I argue that when studying how individuals are affected by and respond to racism, it is methodologically essential to systematically focus on racialised individuals’ sense-making. Only by focusing on sense-making can we understand why individuals respond to racism as they do. Moreover, such a lens helps to explain in-group differences and inspires productive questions about other dimensions beyond race, and their intersections, that might explain these differences.

## Responding to experiences of ethnoracial exclusion

For what follows it is helpful to briefly define two terms that are used (often interchangeably) in the literature on anti-racist responses: “Experiences of ethnoracial exclusion” are defined as experiences of exclusion based on “racial status, ethnicity, national origin, and/or other ascribed characteristics’ (Imoagene, 2019, p. 265; see also Lamont et al., 2016, p. 7). It follows from this definition that experiences of ethnoracial exclusion include (but are not limited to) “experiences of racism,” understood as experiences of being othered, excluded, or discriminated against on the basis of biological or cultural characteristics (see Balogun and Joseph-Salisbury, 2021). Importantly, the focus on experiences and responses to these experiences, whether experiences of ethnoracial exclusion or racism, places the subjective perspective of those affected by these experiences at the centre of attention. The field studying such experiences and individuals’ and groups’ responses to these experiences is ever-growing and has provided insights into individuals’ and groups’ responses in a variety of contexts such as the US (e.g., Lamont et al., 2016; Imoagene, 2018), Brazil (e.g., Moraes Silva and Reis, 2012; Lamont et al., 2016), Israel (e.g., Mizrachi and Herzog, 2012; Guetzkow and Fast, 2016; Lamont et al., 2016), Poland (e.g., Jaskulowski and Pawlak, 2020; Balogun and Joseph-Salisbury, 2021), Germany (e.g., Witte, 2018; Yurdakul and Altay, 2023), Norway (e.g., Ellefsen and Sandberg, 2022), the UK (e.g., Imoagene, 2019), France (e.g., Bickerstaff, 2012; Drouhot, 2023) and many others. These studies have discussed a broad variety of responses, such as talking back (to the “perpetrator,” e.g., someone who makes a racist joke), avoiding potentially problematic situations (by, e.g., not going to a club frequented by White people), meeting up with friends to discuss one’s experiences, becoming politically active in an anti-racist social movement, or management of self. Furthermore, most studies propose some differentiation between types or classes of responses, such as the differentiation between situational response strategies versus discursive response strategies (Witte, 2018), or responses in face-to-face encounters versus retrospective sense-making (Ellefsen and Sandberg, 2022). Based on an extensive review of not only the responses discussed in the literature but also of the various (often binarily organised) classifications, Piwoni (2023) has proposed to differentiate, in terms of practice-based responses, between (1) responses to actual incidents of ethnoracial exclusion in the situation as it happened (e.g., talking back, not responding), (2) responses to actual incidents after they have occurred (outside the situation) (e.g., seeking legal assistance to deal with an incident), (3) strategies to cope with the general experience of ethnoracial exclusion (e.g., a “pick-your-battles”-strategy), and (4) strategies for gaining recognition in society (e.g., political activism, climbing the social ladder).

In interview research, all four categories can be explored: Respondents can, for example, report concrete situations of exclusion and recall what they did in these situations (1) or afterwards (2). They can also talk about strategies or “rules” that they follow in their daily lives to deal with (the possibility of getting into) racist situations (3). Finally, they may talk about how they organise their lives more generally in order to gain respect and recognition. And they may share their thoughts on what they think should be done at a societal level to combat racism (4). However, this fourth category is quite broad and may be the most difficult to address in interview research, not least because individuals do not always consciously reflect on some of the strategies they use as being related to their experience of racism.

The empirical analysis in this study focuses on interviewees’ responses in concrete situations (1) and what they said were their “rules” for dealing with racist situations (3). Responses in concrete situations (1) requires further elaboration. Subsuming categories and examples provided in the above studies, and following Koenig’s (2017) suggestion to use Hirschman’s (1970; see also Lamont et al., 2016, pp. 9, 273) classic distinction between exit, voice, and loyalty as guidance, I propose here to differentiate between three types of responses: confrontation (voice), deflation (loyalty), and the leaving of the situation (exit). Confrontation may take various forms: One may address the racism in the situation and confront “the perpetrator”; e.g., one may “talk back” and thus “teach the ignorant” (Fleming et al., 2012, p. 407). This would be a direct confrontation. Another way to confront is to use counter-questions (e.g., “What do you mean?”), irony or sarcasm, or to fall silent, thus showing “the perpetrator” that their comments/actions were perceived as racist. This would be an indirect form of confrontation. Particularly in situations of “everyday racism” (Essed, 1991), which are encounters that are, per definition, familiar, repeated practices that are “normalised” and rendered “harmless” by members of the majority group (see Bourabain and Verhaeghe, 2021, p. 223), confrontation disrupts the mode of the encounter and contributes towards escalation because the other person does not receive the expected, “normal” answer or reaction. Deflation, on the other hand, encompasses responses that avoid such escalation by any means and may involve giving the expected “normal” answer or reaction, and/or ignoring the (implicit or explicit) racism and continuing with the encounter as if nothing had happened. A third option would be to immediately leave the situation (escape, or distance oneself from it), which could be described, following a distinction used in stress research, as a “flight” response (as opposed to “fight”; see, e.g., McCarty, 2016).

The present study aims not only to understand the diversity of interviewees’ responses in the situation as such and their strategies for coping with the experience of racism, but also, and thereby going beyond previous research, sets out to argue that these responses are related to their understandings of concrete experiences and their general ideas of racism and whether they consider themselves to be affected by racism.^3^

## How people understand (incidents of) racism and consequences for methodology

While the question of how individuals (and groups) respond to ethnoracial exclusion and/or racism has been discussed vividly in ethnic and migration studies, the question of how (incidents) of racism are understood or made sense of by those who are affected and/or targeted has received less explicit attention in the field (but see recently Nadim, 2023; Doering, 2024). Certainly, as already noted (Piwoni, 2024a), the literature on responses is sensitive to the sense-making of racialised individuals and groups, discussing response categories such as “deemphasising” (Witte, 2018), “retrospect sense-making of negative experiences” (denying significance, and talking down) (Ellefsen et al., 2022), “normalisation” (Jaskulowski and Pawlak, 2020) or “ignoring” (Ellefsen et al., 2022; Lamont et al., 2016). However, these response categories are introduced alongside and as situated on the same level as more practice-based categories such as confronting in the situation as such by, for instance, “striking back” (see Witte, 2018). Thus, these studies do not focus on the relationship between understanding/sense-making and practice-based responses, nor do they ask how individuals’ understandings of particular incidents and racism more generally relate to the type of response they choose in a particular situation.

Essed (1991), however, one of the first researchers to study responses to racism from the perspective of those who are affected by it, and who also coined the term ‘everyday racism’, put in her field-defining work *Understanding everyday racism* a strong emphasis on how individuals understand racist events and racism more generally. By introducing the term “comprehension of racist events,” Essed highlights that racist events can, or may not, be understood as racist. Essed’s empirical material are 55 “nondirective” interviews with highly educated Black women, and by delving deeply into their narrations, she argues that individuals follow a “sequence of interpretive steps” so that they can “determine whether a specific event potentially has racist implications or consequences” (Essed, 1991, p. 79). Moreover, and with regard to interviewing as a methodology, she highlights that interviewees’ accounts of racism reflect the process by which they interpret and evaluate racist events: “accounts of racism are not *ad hoc* stories. They have a specific structure based on rational testing and argumentation.” (Essed, 1991, p. 120). Essed (1991, pp. 9, 76–77, 81) also points out that this evaluation takes place against the background of an individual’s knowledge of racism, which is a “special form of political knowledge,” which can be acquired through formal education but also informal channels. While Essed’s work is strongly influenced by cognitive theory, scholars adopting an affect-theoretical and emotional-sociological stance have focused less on “rational testing” and “interpretive steps” but have pointed to the circulation of affects in racialisation processes and racist events (see, e.g., Ahmed, 2004; Tembo, 2021; Bonilla-Silva, 2019; see also Piwoni, 2024a). As Bonilla-Silva (2019) has pointed out, racism is often felt but not rationally understood.^4^ This has important methodological implications: Instead of focusing only on incidents and experiences that individuals clearly and themselves understand as incidents of exclusion, racism or discrimination, researchers should also pay attention to incidents that respondents may not explicitly label as racist or by using comparable labels but about which they may still have and express feelings of unease (see Nowicka and Wojnicka, 2023; Piwoni, 2024a). This is even more so the case in a context as Germany, which can be described as a “culture of racial denial,” which offers only a “limited vocabulary to speak of racism” (Wojnicka and Nowicka, 2023; see also next section). The implication for interview research in particular is that using and focusing only on responses to direct questions about respondents’ personal experiences of racism and/or discrimination may not be very productive, as respondents may shy away from subsuming their experiences under the term racism and/or discrimination. Wojnicka and Nowicka (2023, p. 16; see also Nowicka and Wojnicka, 2023), in their multi-method study in Germany with young people who have a migration history, found that although respondents shared “honest narrations on the rejection, discrimination, and prejudice that participants experienced,” they “were framed without using the term ‘racism’.”

Piwoni (2024a), in a study comparing highly educated Germans of Polish descent, Germans of Turkish descent and Black Germans, found that interviewees talked about incidents and experiences of ethnoracial exclusion in three different ways: (1) by normalisation (interpreting an experience/incident as “normal”), (2) by categorisation (identifying an experience/incident as, e.g., “racist,” “discriminatory,” or “disadvantaging”), or (3) by indicating feelings of unease. Moreover, the experiences talked about in the interviews were not necessarily narrated in response to direct questions about experiences of exclusion but at various other points in the interview. The present study uses the interviews with the same group of Black Germans as in this predecessor study and provides an in-depth analysis of interviewees’ sense-making of both concrete incidents and experiences of exclusion and also of interviewees’ more general reflections on whether they see themselves as affected by racism. It also shows whether and how this sense-making is related to interviewees’ responses to incidents and experiences of exclusion in concrete situations and to what they said were their strategies or “rules” for dealing with racism in everyday life.

## Anti-Black racism in Germany

Germany is an immigrant society with 28.7 per cent of the population having a migration background, i.e., at least one parent born outside the country (Destatis, 2023). It is also currently, after the US, the country with the second largest immigration rate (IOM [International Organization for Migration], 2024, p. 26). However, debates that openly and comprehensively address the existence and prevalence of racism are relatively new.

In fact, for many decades in German public and academic discourse, the term racism [in German: “Rassismus”] itself was avoided because of the atrocities committed under the racist ideology of Nazi Germany and the notion that the term racism should therefore be reserved for explicit and exceptional phenomena of violence. If at all, racism was seen as a problem of a far-right extremist minority (see DeZIM [Deutsches Zentrum für Integrations- und Migrationsforschung], 2022, p. 18). Moreover, as the years-long struggles for and against the renaming of streets in Berlin’s Afrikanisches Viertel (African Quarter) that bear the names of perpetrators of German colonialism demonstrate, acknowledging Germany’s colonial past is not only highly controversial in Germany, but is often discussed without acknowledging contemporary racism (see Barwick-Gross and Kulz, 2024). As pointed out in a report by the *UN Working Group of Experts on People of African Descent* (United Nations, 2017, p. 10), “historical facts concerning the period of colonisation, the transatlantic trade in Africans, enslavement and the genocide of the Ovaherero and the Nama peoples are not sufficiently covered in all schools” contributing “to the structural invisibility of people of African descent in Germany.” Also contributing to the invisibility of Black people is the fact that the actual number of Black people living in Germany is not known because the German census does not allow people to identify themselves as ‘Black’. The number is estimated to be over 1 million, and Black people have typically had one of three ancestries: African immigrants, European soldiers of African descent, or African American soldiers (Aikins et al., 2021, p. 57; Hubbard and Utsey, 2015).

Black movements began to raise awareness of Black life in Germany as early as the 1980s (see Oguntoye et al., 1986), and contributions such as Kilomba’s *Plantation Memories* (2008) and El-Tayeb’s (2020) analysis of the exclusion of Black Germans in between 1890 and 1933 were important in raising awareness for both the everyday racism faced by Black people in Germany and the ways in which racism informed the construction of German national identity in the newly founded nation-state at the end of the 19th century (see also Schönwälder, 2004). However, it has been only in the last decade, and especially with the rise of the Black Lives Matter movement, that broader debates about racism have become more virulent in German society as a whole (see Zajak et al., 2023). In parallel with increased social awareness, policy-oriented research on racism in the German context has provided valuable empirical insights. The study *Racist Realities* [in German: “Rassistische Realitäten”] (DeZIM [Deutsches Zentrum für Integrations- und Migrationsforschung], 2022), which documented for the first time in a representative survey the general German population’s perception of racism as a social reality, showed that although 90 per cent of respondents believe that racism exists in Germany in general, there is a strong tendency to “externalise” racism. 35.1 per cent (tend to) agree that racism is a problem primarily to be found in the USA, and almost 60 per cent (tend to) agree that racism is primarily to be found among right-wing extremists (DeZIM [Deutsches Zentrum für Integrations- und Migrationsforschung], 2022, p. 81). The study also showed, through vignette experiments, that antisemitism and anti-Black racism are more easily recognised in relation to specific situations, such as a comedian cracking a joke involving Jews or Black people, than, for example, anti-Slavic or anti-Asian racism (DeZIM [Deutsches Zentrum für Integrations- und Migrationsforschung], 2022, pp. 63–78).

In addition, the *Afrozensus* (Aikins et al., 2021), the largest survey to date on the realities of Black life in Germany, with 5,793 participants, provides rich insights into the lived experiences of racism of Black and African or Afro-diasporic people in the German context. Although the results are not representative, they show that Black people in Germany experience racism in a wide range of areas of life, from the health care system, where Black people may receive poorer treatment or have their symptoms not taken seriously, to the housing market, where 91.2 per cent of all respondents said they were either very often or often discriminated against (Aikins et al., 2021, p. 90), the highest figure of any area. Not least because it highlights the affective and emotional consequences of experiencing racism, such as mistrust, frustration or even mental illness (see Aikins et al., 2021, p. 239), the study is an important document of the actual reality of anti-Black racism in the German context and its massive significance for those who experience it.

## Data and methods

This study draws on data from 21 semi-structured interviews with Black Germans that I conducted between 2018 and 2021 (see also Piwoni, 2024b, which is based on the same set of interviews, but analyses them from a different theoretical perspective).^5^ It aims at answering the following research questions: (1) How do interviewees understand specific experiences of ethnoracial exclusion, and what is their general understanding of racism? (2) What responses do interviewees say they gave in specific situations (and why), and what are their behavioural strategies? (3) Are there patterns in the relationship between understandings and responses? (4) Are there different types of respondents in terms of how they say they understand and respond to specific experiences of exclusion and racism more generally? In what follows, I outline first the characteristics of the interviewees and the rationale behind the sampling strategy. Second, I explain my methods of data generation (recruitment methods and interviewing questions) and reflect upon my positionality as interviewer. Third, I describe the methods I used to analyse the interviews.

### Characteristics of the interviewees

The sample on which I draw in this article is part of a larger comparative study of three groups of Germans of migrant background (for a description of the data and methods of data collection for this larger study, see Piwoni, 2024a). Of the 21 Black Germans I interviewed, 11 were male and 10 were female, and all interviewees lived either in the cities or metropolitan regions of Hamburg or Frankfurt on the Main. Nine interviewees had at least one parent who had been born outside Germany, while 12 interviewees had two parents who had been born outside Germany (in two cases one parent was born in another European country, and in one case an interviewee had been adopted by White Germans at a young age); they themselves were either born in Germany (second generation; 18 interviewees), or had come to Germany at a very young age (1.75 generation; three interviewees). I identify the interviewees as Black Germans, although I am well aware of what Lukate and Foster (2023, p. 342) call “the context-dependency of racialized identity performances.” Indeed, many interviewees in this study highlighted this contextuality themselves when referring to situations in which they felt, for example, that being from Hamburg was more important than being Black. At the same time, when asked at the beginning of the interview and after we had talked about their (family’s) migration background, how they would identify themselves, many interviewees chose either ‘Black’ or ‘Afro-German’, while some also used labels such as ‘Eritrean German’. Overall, and importantly, by referring to the interviewees as Black Germans, I situate the research within the literature without claiming that being Black German is their permanent and stable identity (for a similar argument see also Lukate and Foster, 2023, p. 343).

The group of interviewees can be seen as belonging to the educated “middle class,” including those with tertiary education, who are typically professionals or managers (see Lamont et al., 2016, p. 289). They all had either an MA or BA (or equivalent) degree or were studying for one, often part-time while working (seven interviewees); two were PhD students at the time of the interviews. The youngest interviewee was 21, the oldest 44, 13 respondents were in their twenties and seven in their thirties. The average age of the 20 respondents who gave their age was 29.2.^6^ Table 1 gives an overview of the interviewees. Note that pseudonyms are used throughout the manuscript.

**Table 1:** Characteristics of respondents.

No.	Pseudonym	Gender	Generation (age of arrival in Germany)	Citizenship	Parents’ countries of origin	Age	Educational status	Occupation
1	Ayi	m	2nd	German	Ghana (father), Germany (mother)	32	MA	Business manager
2	Frank	m	2nd	German	Ruanda (father), Baltic state (mother)	28	Equivalent to BA	Banking professional
3	David	m	2nd	German	Eritrea (father), Eritrea (mother)	33	MA	Business manager
4	Benard	m	1.75th (3)	German	Ghana (father), Ghana (mother)	44	Unknown	Consultant in the non-profit sector Student
5	Dan	m	2nd	German	Eritrea (father), Eritrea (mother)	31	MA	Trade union official
6	Irina	f	2nd	German	Ruanda (father), Baltic state (mother)	31	BA	MA Student
7	Linda	f	1.75th (2)	German	Adopted, biological parents from Liberia	31	MA	Researcher
8	Ada	f	2nd	German	Senegal (father), Germany (mother)	31	MA	Teacher
9	John	m	2nd	German	Gambia (father), Germany (mother)	27	Highschool (Gymnasium)	Activist Student
10	Yohanna	f	2nd	German	Eritrea (father), Eritrea (mother)	29	Equivalent to MA	Doctor
11	Miriam	f	2nd	German	Senegal/Burkina Faso (father), German (mother)	21	Highschool (Gymnasium)	Workshop organiser, actress BA Student
12	Iza	f	2nd	German	Eritrea (father), Eritrea (mother)	39	MA	Influencer, teacher
13	Vian	f	2nd	German	Nigeria (father), Germany (mother)	26	BA	Banking manager
14	Amma	f	2nd	German and Ghanian	Ghana (father), Ghana (mother)	end 20s	BA	Marketing manager (part-time) MA Student
15	Sara	f	2nd	German	Eritrea (father), Eritrea (mother)	29	MA	IT Manager
16	Tani	f	2nd	German	Nigeria (father), Germany (mother)	25	MA	Researcher PhD Student (MINT)
17	Amar	m	2nd	German	Senegal (father), Germany (mother)	27	BA	MA Student
18	Napo	m	1.75th (2)	German	Togo (father), Togo (mother)	25	BA	IT Professional MA Student
19	Elias	m	2nd	German	Eritrea (father), Eritrea (mother)	26	MA	Architect
20	René	m	2nd	German	Nigeria (father), Germany (mother)	24	MA	Researcher PhD Student (MINT)
21	Daniel	m	2nd	German	Germany (father), Ethiopia (mother)	25	BA	MA Student

Type 1 interviewees are highlighted in dark grey, and Type 2 interviewees in light grey. The four respondents who did not fit either type are not highlighted.

The rationale behind the sampling strategy was to focus on individuals who are structurally assimilated in terms of formal status (all interviewees held German citizenship and one interviewee held dual citizenship), knowledge of the German language, educational attainment, and/or integration in the job market, but who may still get confronted with racism in their daily life. According to the literature on the “integration paradox,” the economically more integrated and relatively highly educated immigrants may turn away from the host society, not least because they perceive more discrimination, which may be explained by their greater consumption of host country news media and greater contact with members of the mainstream society, and thus greater exposure to discrimination and exclusionary messages (see Schaeffer and Kas, 2024). Therefore, the respondents in this study may have been particularly inclined to recount experiences of exclusion, given their educational background. However, as the results also show, even within a group of highly educated respondents, the understanding of experiences can vary widely.

### Methods of data generation

To recruit interviewees, I used multiple points of entry, including personal contacts, professional networks such as LinkedIn and Xing, special interest organisations such as ADAN e.V.,^7^ Facebook ads, and also a professional recruiting firm. Additionally, I employed snowball sampling. Before the interviews, I obtained informed consent and all participants were assured of confidentiality and anonymity. The research process was conducted according to guidelines approved by the ethics committee of the University of Passau (approval number 07.5095). The interviewees recruited through the professional company received compensation for their participation, while others were offered 10€ vouchers for online (book-)stores. However, not all interviewees accepted the vouchers and, in some cases, I refrained from making the offer to avoid potential discomfort.

Interviews were described in advance as focusing on “everyday experiences” and “identity” and were done either by phone or (VoIP)-mediated technologies (Skype or Zoom). Online communication has become extremely common for members of the middle class, and I was able to establish rapport and generate trust with the interviewees (for similar experiences with regard to the advantages of Skype- and Zoom interviewing, see, e.g., Archibald et al., 2019; Żadkowska et al., 2022). All interviews were conducted in German. They lasted between 45 min and 160 min (average length 90 min) and were afterwards transcribed verbatim.

### Interview questions, interviewing style, and interviewer’s positionality

The interview questions were designed as open questions to elicit extensive accounts and narratives. I always started by asking the interviewee to provide some information about themselves in terms of age, occupation, citizenship status, and how long they have been living in Hamburg or Frankfurt, respectively. We then turned to talk about the interviewee’s self-understanding and feelings of belonging and whether (or not) and in which situations they felt their migrant background mattered in their daily life. I also explicitly asked whether they had made any experiences of exclusion, such as getting discriminated against. In cases when interviewees had introduced the term “racism” themselves, I picked the term up in my wording of the question of experiences of exclusion. Overall, the interviewing style was receptive, in that interviewees had a large measure of control in answering the relatively few questions I asked (Brinkmann, 2013, p. 31).

It is important to keep in mind that what interviewees share in the interview situation—experiences, attitudes, accounts of what strategies they follow in their lives—are not facts per se, but co-produced (with the interviewer) in the interview situation (see Holstein and Gubrium, 1995). In qualitative interviews, we as interviewers cannot help but always enter into conversations and participate in meaning-making practices (see Brinkmann and Kvale, 2015, pp. 181–184). As a result, there is a need to reflect on our positionality and how it may have influenced the interviews.

I am a middle-aged German mother whose parents immigrated from Poland to Germany, a fact that I revealed to the interviewees at the beginning of the interviews. Although I shared a background of migration and a comparable class and generational position with the interviewees, they may have perceived me as a White person and therefore as not sharing the same experiences of racism as Black people do in the German context. Although it has been argued that immigrants from Eastern Europe are positioned “on the peripheries of whiteness” in that they are “both racialised and able to benefit from their position as ‘paler migrants’” (Narkowicz, 2023, p. 1534), most interviewees differentiated between “visible” and “invisible” immigrants and assumed that they would make different experiences than White persons of migrant background in their everyday lives. A few interviewees also asked me at some points of the interview, and typically after sharing a specific episode or type of experience (such as being asked the “Where are you from?” question), whether I had similar experiences. Overall, and despite my migrant background, interviewees did not seem to assume that I knew “what things are like” for a Black person in Germany, which may have been conducive to them engaging in longer narratives, explication and detail regarding particular episodes of ethnoracial exclusion. At the same time, I felt that my role as a scholar was rather conducive to them sharing their experiences, understandings and reflections given their educational status. Many interviewees also said that they gladly contributed to research projects in general, while others pointed out that they were supportive of projects drawing academia’s (and the general public’s) attention to the experiences of Black people in Germany. This may, of course, have led them to talk about their experiences in a way that they felt was appropriate to achieve these aims. Furthermore, the fact that I am White (albeit from a migrant background), combined with the fact that I waited to use the term “racism” until the interviewees themselves brought it up, may have given some of them the impression that I was suspicious of their experiences of racism and that I represented the German culture of denial of racism.

However, and as the findings demonstrate, interviewees’ narratives were honest and rich, and it is thus plausible to assume that the understandings and responses identified were part of the interviewees’ “cultural repertoires” on which they regularly drew on outside of the interview situation, too (see also Swidler, 1986).

### Methods of data analysis

I used MAXQDA software to analyse the interviews. The analysis combined deductive analysis (examining the data through the “lens” of specific concepts) and inductive analysis. Below I describe the analytical process, which involved five separate steps, in relation to the four research questions above.

In a first step, I identified all experiences of ethnoracial exclusion that interviewees told me about. For purposes of identification, I drew on the concepts of “experiences of stigmatization” and “experiences of discrimination,” with the former including experiences where individuals had experienced “disrespect and their dignity, honour, relative status, or sense of self was challenged” including “instances where one is stereotyped as poor, uneducated, or dangerous, or where one is misunderstood or underestimated” (Lamont et al., 2016, p. 7) and the latter including experiences of being “prevented [from] or given substandard access to opportunities and resources such as jobs, housing, access to public space, credit, and so on because of their race, ethnicity, or nationality” (Lamont et al., 2016, p. 7).^8^ More specifically, I included experiences that interviewees shared in direct response to my question about experiences of exclusion, as well as experiences shared at other points in the interview. I also counted all the experiences mentioned by interviewees. Secondly, and thereby addressing the first research question about how interviewees understood specific experiences of ethnoracial exclusion, I first analysed and then coded within a closed coding frame how interviewees made sense of these experiences in terms of categorisation (labelling an incident), feelings of unease (by, e.g., indicating affects of unease/ambivalence regarding the experience), or normalisation (framing an experience as “normal”) (for further details regarding this step, see Piwoni, 2024a). Additionally, I analysed, for each interviewee, whether at all, and if so, how they talked about discrimination and racism in general during the interview (e.g., whom they believed was affected by racism, how severe they believed this was a problem in German society). In a third step, in order to answer the second research question on responses and strategies, I analysed how interviewees said they had responded to the specific experiences narrated in the interview (note that this was not always reported). While this step was informed by the categories of responses that are discussed in the literature (see above), this step resulted in an inductively generated unique classification that distinguishes between direct confrontation, indirect confrontation, flight/exit, and continuation of mode of encounter/deflation. In addition, I analysed which behavioural strategies respondents reported using in their lives to cope with exclusion. Fourthly, and thus addressing the third research question about patterns in the relationship between understandings and responses, I used the constant comparative method (see Boeije, 2002) and compared all the experiences reported and especially how interviewees made sense of them (in terms of categorisation, feelings of unease, or normalisation) on the one hand, and interviewees’ reported responses on the other to see if certain understandings and responses typically occurred together. Fifth, and concerning the fourth research question about types of interviewees, I compared interviewees in terms of their understandings of and responses to experiences and racism to understand whether there were different types of interviewees.

The main findings are presented below and by way of introducing two types of interviewees. All quotations are taken from the transcripts, which have been translated from German into English and in some cases edited for clarity or context. Interviewee pauses (‘…’) and omissions in quotations (‘[…]’) are used where appropriate.

## Findings

As outlined, interviewees narrated experiences of exclusion at several points in the interview and not just in response to the direct question about whether they have experienced discrimination and/or racism. These experiences included both experiences of stigmatisation and of discrimination. Most often, however, interviewees recounted experiences of stigmatisation, which is a common finding in many studies of experiences of exclusion (see, for example, Lamont et al., 2016; Imoagene, 2019; Witte, 2018). For example, interviewees talked about being asked “Where are you (really) from?,” being “praised” for their German language skills, having all kinds of comments made about their hair, and also having their hair touched without asking. Experiences also included racist jokes, outright insults, verbal abuse and being confronted with stereotypes about Black people or people “from Africa” including exoticisation. In addition, interviewees often mentioned facing double standards and being treated unfairly both at school and at work. Many had also experienced being undervalued, their qualifications doubted, or being called an “exception.” Male respondents, in particular, reported being frequently stopped by the police and denied entry to nightclubs. And several interviewees mentioned that their primary school teacher had refused to recommend them for the Gymnasium, an upper secondary school in Germany. Overall, and when compared to what respondents had reported in the Afrozensus (Aikins et al., 2021), the experiences mentioned by interviewees in this study appear to be fairly typical of (relatively highly educated) Black people’s experiences of racism in the German context (note that respondents with higher education were over-represented in the Afrozensus; see Aikins et al., 2021, p. 68).

I found that across 21 interviews, interviewees reported a total of 246 experiences of exclusion. This number includes 195 experiences that interviewees said they had experienced themselves and 51 experiences that interviewees said others (family members, partners, etc.) had experienced. Thus, on average, each interviewee reported about 9–10 experiences that they themselves had experienced. However, while some respondents reported many such experiences (up to 17), others reported few (no more than three). I also found that these differences were related to how interviewees made sense of their experiences and how they tended to respond to them. In what follows, I will present two types of interviewees representing 17 of 21 interviewees. These two types stand out because they contrast in all four dimensions that this analysis explored. Most importantly, they contrast in terms of their knowledge of racism and how they make sense of experiences of exclusion, and are therefore ideal for exploring in detail and depth how different understandings of racism and racist incidents relate to the responses given in such incidents. Importantly, the description of these two types should not be taken to imply a value judgement about which understandings and responses (or: response strategies) are more valid and/or effective. The four interviewees who did not fit either type are discussed in the discussion and conclusions section of the article.

### Type 1: “[T]o me, some kind of racist incident happens twice a month”

Twelve interviewees narrated not only many incidents and experiences of exclusion in past and present, but also, and in relation to most of the incidents, did not hesitant to frame these experiences as instances of racism. In fact, many Type 1 interviewees told these incidents in direct response to my question about whether they had experienced exclusion but also at other points in the interview and in some cases even at the very beginning of the interview and unprompted—e.g., when reflecting on how they would describe themselves and their feelings of belonging (arguably, these questions are often intertwined with the experience of being othered; see, e.g., Creese, 2019). Overall, Type 1 interviewees felt they were constantly and potentially always affected by racism; as Linda said: “to me, some kind of racist incident happens twice a month.” A typical way of presenting their experiences was within a longer thread of memories as in Ayi’s case, when he responded to my question about whether he had had racist experiences.

Yes, starting in infancy with “Can I touch your hair?” and so on. Then with stupid comments. There was a situation once when I was on a parent-child health retreat with my mother, and I was teased so much because of my skin colour that at one point I just wanted to stop the retreat, which is actually the opposite effect of a retreat. […] Exactly, in primary school a little bit, but that actually worked. In secondary school, I would say hardly at all. […] At least I don’t remember it. And then it started again, where it was about going out and clubs and so on. That’s when you really felt it again, [problems] with getting in. Exactly, everybody wanted to be in the queue with the White people in the group to have a chance of getting in. […] And then the marginalisation […] like at a party and I’m the only one asked where I’m from or something, just that kind of marginalisation.

Interestingly, many Type 1 respondents said that as children and teenagers they had not been able to understand the workings and many different facets of racism, but still “knew” or “felt” that something was wrong, when, e.g., they were confronted with a racist slur. Iza shared a memory of how she felt when she was chosen out of all the children in the class to read a passage that contained the N-word.

No one has told us Black people that the N-word is an insult to you. Yet we all Black people feel the same. Isn't that strange? […] How did I know when I was a little kid that it was an insult? […] We read a lot of stories [at school], nothing with the N-word. Suddenly I’m supposed to read and then the N-word comes up. That was the end for me, I was totally exhausted. But the teachers didn’t care, they just didn't care. They just carried on. They saw how sad I was. They didn’t care. And that’s something I’ve remembered.

As well as recounting many episodes of unfair treatment (at school, at university, when applying for jobs), Iza said that although she felt that something was “not right,” she did not know what to call it or how to make sense of it. It was only later that she learned to make sense of these experiences.

I worked as a stewardess for a year. […] And then I just flew around. And yes, I got to know some Black women and they told me a bit about racism, about discrimination. And then at some point I realised that it was the same for me.

Iza’s description of how she eventually became aware of how racism works is typical of how many Type 1 interviewees described their biographies—(only) now, they said, were they able to fully understand racism and its many manifestations.^9^ Many interviewees in this group had worked to build their knowledge about racism by meeting other Black people, as in Iza’s example above, who made them aware that other Black people also experience racism on a regular basis. They also said that they had increased their knowledge through books such as *Exit Racism* by Ogette (2020) or *How to be Black* [Anleitung zum Schwarz sein] by Chebu (2014), and by joining networks such as ADAN [Afro-Diasporic Academic Network] or ISD [Initiative for Black People in Germany] and attending their workshops and/or meetings. As a result, some of these interviewees had a pronounced knowledge of the nature of racism such as that it is also a structural, systemic, and institutional problem, that it occurs both covertly and overtly, or of certain stereotypes about Black people, and also of Germany’s colonial history (see also Essed, 1991, pp. 105–118).

Nonetheless, most Type 1 interviewees did not narrate all incidents of exclusion by ways of clearly categorising them as “racist,” “discriminatory,” “exclusionary” or similar. Sometimes, despite their broad knowledge of racism, they were unsure and doubtful about how to make sense of a particular situation or someone’s behaviour and thus expressed feelings of unease. There were also specific classes of incidents, such as the “Where are you from?” question (or positive racism, when receiving “compliments”), which some Type 1 respondents were reluctant to explicitly frame as exclusionary, but which they reported by indicating or describing feelings of discomfort in relation to the incident (see Piwoni, 2024a,b).

How did Type 1 interviewees say they would respond to incidents of racism? Very often interviewees said that such incidents were accompanied by strong emotions on their part, which made them either “fight” or “flight.” Especially in situations where they felt in (physical) danger, such as when they were attacked by skinheads, interviewees said that they tried to get out of the situation as quickly as possible. However, trying to get out of a racist situation was not a strategy limited to physically dangerous situations. Some interviewees used this response more generally and whenever they were confronted with racism. For example, Dan, who reflected on how, when confronted with everyday racism such as being asked “Where are you from?,” he said that he felt an impulse to “get out of this situation so quickly.”

Alternatively, “fight,” in the sense of “confront,” or “call out/address racism” was the response (option) that Type 1 interviewees said they most often considered and often felt an impulse to choose (but ultimately did not always choose). An extreme example of the affects triggering a literal “fight”-response was narrated by Dan, who shared the following memory:

And we just went for a walk. […] And Grandma pushed the pram. And I held the umbrella, it was raining, over our heads. And then a guy came by, somehow [on a] bicycle. And then he started calling me the worst kind of racist names. He also said the N-word several times […], and then I thought to myself: Huh? That’s… And then I briefly thought about punching him in the face and so on…. And I even ran after him for a moment. But then I realised: Okay, I can’t do that now.

Other interviewees reported racist situations in which they verbally confronted the perpetrator. As an alternative to directly calling out racism, some interviewees said that they used questioning, sarcasm or silence as indirect ways of confronting the other person and indicating that they had understood the racist nature of the incident. As Miriam elaborated:

Yes, I do have a strategy. And that is […] to ask counter-questions, like “Why?”. “What do you mean? What do you mean now?”, blah, blah, blah. I always do that. And then most people quickly find themselves in need of an explanation […]. And then they get really nervous really quickly. And if a person gets nervous quickly, then I’m like: “then you’ll realise that you’ve done something wrong; then you don’t need to tell me anymore”, in that sense. And that’s actually the most empowering strategy, because then you don’t have to explain why someone is nervous, they have to figure it out for themselves.

However, did Type 1 interviewees did not always (choose to) confront (either directly or indirectly), and, overall, they gave four main reasons for not doing so: Firstly, because they were “in shock” and therefore unable to react immediately; this non-reaction was sometimes later accompanied by feelings of regret for having been passive. As Miriam said:

But with things that are […] racist, you’re always so perplexed at first and so surprised at this impertinence that you sometimes miss the moment to react, I have the feeling. And then you always end up saying “Oh, I should have said that and I should have done that” and you’re really unhappy with yourself.

Secondly, some said that they did not confront because they were generally trying to “go high” and make a positive impression by example as a more effective long-term strategy to combat racism. Dan, at another point in the interview, said that he now subscribed to “respectability politics”:

So, to always present the best image of yourself […]. Even if you’re facing blatant hostility and so on. And then just always show this best image, so to speak. […] To say: “Yes, everything bounces off like Teflon. And you can’t do anything to me.” And: “I’m taking the high road.”

Thirdly, Type 1 interviewees said that they did not always confront because of a “pick your battles” strategy (often accompanied by a sense of general fatigue). Frank articulated this strategy and the reasons for choosing it as follows:

I’ve become a little tired in the meantime. To be honest. I wish I would always do that [address racism directly]. But I know that I often just don’t have the strength to do it. I see something [a racist incident]. I know it [that it’s racist]. And I think to myself: I can’t deal with it now. I’m not proud that it’s like that. But it’s just become that way. I think a lot of people who fight against discrimination feel that way. […] At some point you just have to see which battles you want to take part in. And which ones you just have to let pass by out of self-protection, I’d say. […] [It] is very exhausting. It’s often one person against the majority. It’s, well, as a minority you rarely have any support at the moment.

Fourthly, some interviewees said that they considered several aspects such as the severity of the incident, the importance of the setting in which the racist incident occurred, and the relationship they had with the “perpetrator” (see also Bickerstaff, 2012). In the workplace, for example, some respondents said that they were more reluctant to confront racism directly. Like Linda, who said:

I always differentiate according to where I am. Am I in a professional setting, for example at work? Is it in a private space? I make a very clear distinction here. At work, I’m definitely calmer and don’t get into so many discussions because I don’t have anything else to do with people.

As Wingfield (2010, p. 256) noted in her study of Black professionals in the US, in many professional settings, workers are expected to express “pleasantness and congeniality as important feeling rules,” which puts Black people, who often face racism in the workplace—“in the form of racialized comments, stereotypes, and beliefs from colleagues”—, in a difficult position. And yet Wingfield’s interviewees tried to meet these behavioural and emotional demands. Although Linda points to her lack of closeness to her colleagues as a reason for not directly confronting racism in the workplace, organisational cultures such as those outlined by Wingfield (2010) may also be relevant in the German context.

Finally, some respondents also said that it was simply not “their thing” to confront. As Yohanna explained:

I have to say, I’m not a person who likes to lecture other people, who is always looking for some kind of confrontation, but I just prefer to look for an environment in which I feel comfortable […], where you simply feel in good hands and don’t have to explain yourself all the time.

So in cases where respondents did not leave the situation or choose to confront, what were the alternatives for responding to incidents of racism? Many respondents said that they either ignored the racism or responded in a humorous but non-confrontational way. In doing so, they responded in a way that allowed the encounter or situation to continue or end smoothly. Yohanna, for example, reported the following incident as part of an extended narrative of how she understood comments about her hair to be racist:

So now, for example, I braid my hair from time to time. And then it's suddenly 20 centimetres longer, which of course makes it clear that I have extensions in it. That was recently, when a colleague […] commented on whether I had extensions in there, which is very obvious because my hair can't grow that quickly overnight. And then I said: ‘No, they grew overnight.’ So, just… […] Well, people usually laugh then too. I say it with a laugh so as not to make any enemies at work. But it's also obvious that […] that was a stupid question.

Here, Yohanna explains that she did not want to make enemies at work, so she chose a non-confrontational but still not entirely passive response because she found such comments problematic and racist. In particular, Yohanna’s aim here is to make it clear that “it was a stupid question,” but not to call out the racism underlying her colleagues’ comments about her hair. Of course, such responses walk a fine line between indirect confrontation and a deflationary response that allows the mode of encounter to continue. Yohanna also said at another point in the interview that she often just gives the “expected” response, such as when asked where she is (really) from, and she said that she often just ignored racist comments, jokes, stares or other incidents. Such responses are of course “safer” in terms of deflation than a humorous response.

Type 1 interviewees were most likely to recognise racism in its various manifestations. They were also the most reflective and aware of their responses and response strategies. Although recognising racism was associated with strong feelings and a state of being affected by the situation as such, and in some cases led respondents to immediately confront the perpetrator or to flee the situation, most Type 1 respondents said that they had come to make conscious choices about how to respond, often following a specific, overarching behavioural strategy. Thus, although confrontation was within the “repertoire” of response options for all Type 1 respondents, they often chose not to confront.

### Type 2: “I wouldn’t say that it was racism”

In stark contrast to Type 1 interviewees, Type 2 interviewees (5 out of 21) not only reported very few incidents of exclusion, but also tended to either normalise these few incidents or to present them as “exceptions.” For example, Sara, who was convinced that she had never been denied a chance or had been treated unfairly because of her being a Black person, described her experiences at school as “okay” but also remembered:

But what I noticed was that […], in primary school, I was advised to go to Realschule [secondary school] rather than Gymnasium [upper secondary school]. It wasn’t that my grades were super bad, but they weren’t super good either. They were in the satisfactory range and I realised that others with similar grades could of course go to Gymnasium. I was told “You probably won’t”, so I was very sad. […] but I wouldn’t say that it was racism….

It is well documented that in Germany children from migrant backgrounds are underrepresented in the “Gymnasium”, an upper secondary school that prepares students for university (see, e.g., Gogolin et al., 2019). This can be explained by a number of factors, including discrimination in grading (see, e.g., Sprietsma, 2013). However, Sara was reluctant to describe her experience as an instance of unfair treatment, although the very fact that she shared this memory suggests that she was (and still is) unsure how to make sense of it.

Daniel, a 25-year-old student with an Ethiopian mother and a German father, responded to my question about whether he had experienced exclusion or unfair treatment with “no, not necessarily directly.” He continued with a train of thought, stopping himself at several points to confirm that he had not been treated unfairly. However, he also said he felt that he was perceived “differently” but was also unsure: “because I always ask myself the question, okay, does that also have to do with my self-image or with my external image, and do I interpret too much into some looks or some interactions.” He also mentioned a period in his life, “two or three years ago,” when he was stopped by the police “several times” while driving his car:

Then I asked myself what could be the reason for that? Whether it was because I was behind the wheel or something, because that was much more often than I had heard from all my friends, more often than they had been stopped in their entire time as drivers. And exactly, but apart from that… actually rather less. So, I haven’t personally experienced it [being treated unfairly] that much, I have to say, fortunately.

Although the fact that he had been stopped by the police significantly more often than his White friends had made him suspicious, Daniel refrained here from using terms such as “institutional racism” or “racial profiling”—terms that are increasingly being used in the German mass media (see, e.g., Leitlein, 2020). Daniel was born in Germany but had lived for several years in Ethiopia, where he attended a German school, before returning to Germany in his teens. He often referred to this fact when, for example, he pointed out that he did not have much experience of how to respond to (everyday) racism: “I also lack a bit of experience, I can only speak of luck that I’m not affected like that.” However, it also became clear that he would not frame certain experiences as (everyday) racism that Type 1 interviewees often did, but instead normalised them. For example, the “Where are you from?,” which most Type 1 interviewees clearly understood as othering and racialising, especially in cases where it was the first thing asked (see Creese, 2019), was perceived very differently by Daniel:

So, I think that’s a question where I firstly assume that there is simply curiosity. Personally, I would also describe myself as very curious and perhaps to a different extent, so I wouldn’t ask anyone that directly, as it somehow happened with me. […] So at least I see it as being based on some kind of curiosity, that it wants to stimulate an exchange, along the lines of where do you come from, where do I come from and that you then just talk about it. […] So at least I’ve rarely had the experience, or not at all, that it was in any way pejorative or associated with a, shall I say, non-curious intention. And that’s why I think it’s actually okay.

For Daniel, racism was a question of the intentions of the individual, and he said that in principle he had never experienced anyone with bad intentions. Neither considering systemic, structural or institutional racism, nor the historical roots of contemporary forms and manifestations of racism, Daniel seemed to consider only the possibility of individual, blatant and ill-intentioned expressions of racism—an understanding that was characteristic of Type 2 respondents in general.

What was also very salient in Daniel’s narrative, and typical of all Type 2 respondents, was his reference to being “lucky” not to have had hurtful experiences, combined with a reluctance to identify particular types of incident as problematic. David, whose parents had emigrated from Eritrea, argued along the same lines when he said that many immigrants were “more sensitive,” although questions such as “Where are you from?” were “indeed the most normal thing.” On the other hand, he felt that he was personally very lucky: “I am lucky. Fortunately, I don’t necessarily experience [discrimination]. Or maybe I ignore some things. […] Yeah, so, me personally, I haven’t experienced it [discrimination], but I’m also very liberal, so I don’t get offended easily, and, yeah, […] I try to put myself in every person’s shoes. If you have an older lady, it’s not directly racist if she says something funny. […] She’s just an older lady who grew up in a different world.”

Common to all Type 2 interviewees was a tendency to normalise the relatively few incidents they mentioned, or to indicate only feelings of unease in relation to specific incidents. And as for the few incidents that they would describe as “exclusionary” or “racist,” these were often presented as incidents from their childhood and teenage years (and thus as related to a “different time”) or as exceptions, which they further explained by the provinciality of the perpetrators and/or a lack of knowledge/education (see also Jaskulowski and Pawlak, 2020, p. 461). When discussing incidents of everyday racism, such as being asked inappropriate questions, Sara reflected on the people who asked these questions as follows:

[These are] people [who] haven’t actually seen much of the world unless it comes from the television. And then they also have this fear of new things. That doesn’t mean they’re immediately evil or racist. It just means that people need time to experience something new. To understand it, process it and deal with it.

Overall, Type 2 did not see racism as affecting and permeating society as a whole but rather as a characteristic of certain groups of individuals who deliberately hold racist views, such as right-wing extremists. However, individuals who they believed had no ill intentions were not seen as racist and Type 2 respondents were quick to excuse them.

As a consequence, Type 2 interviewees did not see their own lives as defined by the experience of racism, nor were they quick to interpret situations or individuals as “racist.” As René said:

So, of course it also depends on what you consider racism to be. There are also people who are not sensitised, who […] don’t know that perhaps certain terms shouldn’t be used in this way, who don’t know that some questions are insensitive. I wouldn’t characterise such people as racist.

To explain why they felt relatively unaffected by racism and had made only few experiences of exclusion if any, Type 2 interviewees not only emphasised that they were “lucky” (as shown above) but also came up with explanations such as the multicultural and liberal environment in which they lived and worked. David, for example, had played football as a student in a city other than Frankfurt. There, he had been called the N-word by players and fans of opposing teams several times when playing in smaller towns, without the referee intervening and sanctioning it. In Frankfurt, however, he said he would never experience anything like that. Later in the interview, David asked me if I had heard of the ISD [Initiative for Black People in Germany]. Founded in 1985, the ISD is the oldest and arguably the most experienced community-based organisation of Black people in Germany, with a strong focus on raising awareness of everyday racism, racist violence and police violence.^10^ David found it “important that the association exists” but he also said that he would “lie if I were to join this association now and stand up for it myself, because I just don’t… [have the same experience of being racialised] myself, so I can’t support it. Not supporting is perhaps the wrong word, but I just don’t feel that way because I grew up in Frankfurt. I was born in Frankfurt, it’s like a different world. When people tell me what’s happening in the villages or what’s happening in other cities, I just shake my head.” Sara had an alternative explanation. She believed that because she was a Black women, she had “fewer problems” than a Black man, and “because I can express myself and sell myself to a certain extent, which you have to do when you are looking for a job, or starting a new chapter in your life, so I did not necessarily have these hurdles.”

Overall, Type 2 respondents’ general knowledge of racism was less extensive than that of Type 1 respondents. As Essed (1991, pp. 79–87) argues, a poorly developed general knowledge of racism affects how individuals evaluate racist events. More specifically, a lack of such knowledge can lead to (1) interpreting certain events or practices as acceptable (e.g., in cases of covert racism, such as the question “Where are you from?”), (2) excusing unacceptable behaviour with acceptable reasons (e.g., that people are just interested when they ask you where you are from), (3) not believing that the event happened because you are a Black person (e.g., Sara who did not get a recommendation for the “Gymnasium”), (4) finding mitigating circumstances that make the specific case excusable (e.g., René points out that people simply do not know that some terms are racist), (5) not acknowledging the social relevance of the act by framing being affected by it as a “personal problem” (e.g., David, who says that he is not “easily” offended), and finally (6) not being able to evaluate the event as an instance of racism (all interviewees in this section).

In contrast to Type 1 interviewees, Type 2 interviewees did not see confrontation as an option and they always responded in a non-confrontational way, with the intention of not disrupting the flow of the encounter. In situations of everyday racism, such as being asked inappropriate questions about one’s hair or when being lauded for one’s language skills they would give a polite and informative answer to the questioner. Daniel even saw such situations as an opportunity to make the questioner aware of Germany’s multiculturalism: “But these are all things where I think to myself, okay, maybe you can give a little bit of food for thought or maybe you can help a little bit to create a more objective picture somehow. “He said that he never responded in an ironic, sarcastic or confrontational way, and although he could understand if others were annoyed by, for example, the “Where are you from? “question and responded in a confrontational way, he did not feel that way:

But the flip side of this is that you have to say to yourself, okay, but if someone like me or someone with a migrant background perhaps doesn’t provide information or doesn’t talk about it, how else do people get their information or how else do they somehow get the insight that I personally could provide, for example.

Type 2 interviewees also mentioned ignoring as a response strategy as David noted in the quote above (“Or maybe I ignore some things.”). Here, ignoring should be understood as part of a broader behavioural strategy, that is not intensely reflected upon, rather than as a consciously chosen response to and in the situation itself, in the sense of “this is racism, but I will ignore it” (as part of a “pick-your-battles” strategy, for example). Ignoring here involves an established and ingrained disregard for wanting to make sense of various situations and a choice to generally “switch off” one’s sensors. Indeed, René described his attitude as “stoic” and himself as principally “unaffected” by potentially problematic incidents, and Amar stressed that one of his qualities was that he was “open” and “I don’t feel directly attacked so quickly” and “I can laugh at myself.”

## Discussion and conclusions

This study compared across 21 Black German interviewees similarities and differences in four dimensions: (1) personal experiences of exclusion (number of incidents and interviewee’s assessment of how frequently they experience exclusion), (2) how interviewees made sense of their experiences (in terms of: normalisation, categorisation and feelings of unease), (3) understandings of racism, and (4) how interviewees said they would respond to incidents of exclusion and their overall behavioural strategies.

Two contrasting types of interviewees were identified: First, there were interviewees (Type 1) who not only reported a relatively high number of experiences of exclusion and described having to cope with exclusion regularly, but who also made sense of many of these experiences in terms of “racism.” At the same time, they had a broad and sometimes theoretically informed knowledge of (the workings of) racism. While they adopted a variety of behavioural strategies, of which the “pick your battles” strategy was a very popular one, they also had a repertoire of options at their disposal for responding in the situation itself, ranging from direct and indirect confrontation (by calling the “perpetrator” out on racism, or educating them, using counter-questions, sarcasm, or irony, or falling silent) to leaving the situation, and the option of continuing with the mode of encounter (by ignoring, giving the “expected” response, or a humorous yet non-confrontational response).

**Figure 1:**
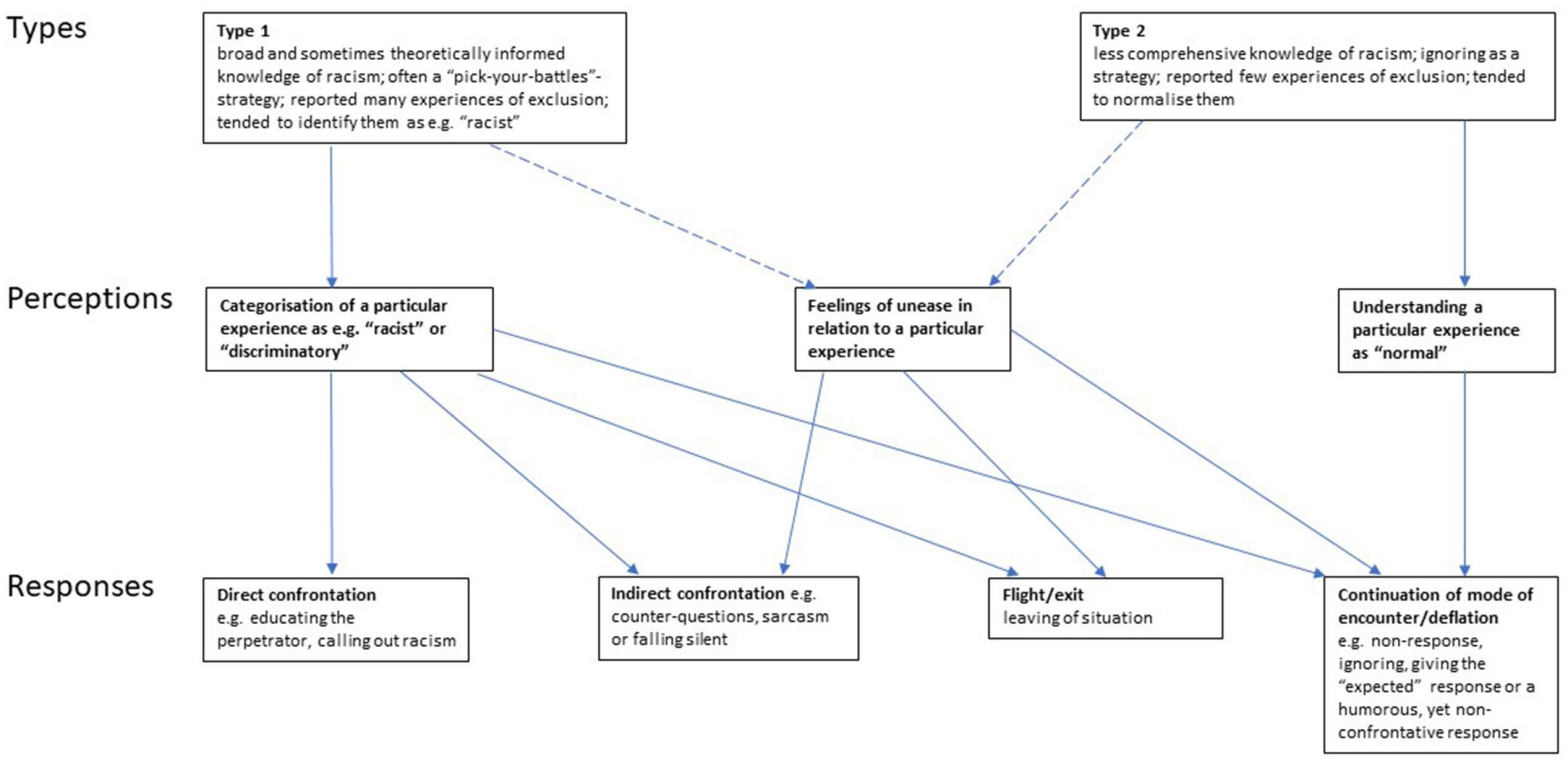
Types of interviewees, perceptions of and responses to experiences of exclusion.

Second, and in contrast to Type 1 interviewees, Type 2 interviewees reported relatively few experiences of exclusion, shied away from making sense of these experiences in terms of “racism,” and tended to normalise many incidents or indicate only feelings of unease. Similarly, they had a less comprehensive understanding of racism, and adopted a different behavioural strategy of ignoring. In incidents of exclusion themselves, they most often reacted by ignoring, giving the “expected” response, or giving a humorous but non-confrontational response. Figure 1 gives an overview of these two types of interviewees and how they typically perceived incidents of exclusion (Type 1 through identification as, e.g., “racist; Type 2 through normalisation; both types also mentioned feelings of unease in relation to specific incidents of exclusion, but less frequently). Figure 1 also shows how the perceptions of incidents of exclusion relate to responses.

At least three conclusions can be drawn from the analysis of the relationships between perceptions of and responses to incidents of exclusion as they appeared in the analysed interviews: Firstly, it is only when an incident is categorised as, for example, “racist” that respondents may choose direct confrontation. It is unlikely that one would respond with direct confrontation to incidents that make one feel uncomfortable, but that one is reluctant to clearly categorise as, e.g., “racist.”

Secondly, perceiving an incident as “normal” will not lead to confrontation, but to a response that is conducive to continuing the mode of encounter, e.g., by giving the “expected” response. Thirdly, understanding an incident as, for example, “racist” can lead to very different responses (from direct confrontation to deflation by continuing the mode of encounter) depending on one’s overall behavioural strategy and other circumstances.

By demonstrating that different understandings lead to different responses and that there are different types of interviewees in terms of their understandings of racism and racist incidents and their preferred responses and strategies, this study argues for the systematic consideration of individuals’ sense-making when studying responses to racism. While research has pointed to variability in how people understand racism and/or racist events (see, e.g., Piwoni, 2024a; Nadim, 2023; Essed, 1991; Lamont et al., 2016), the link between these understandings and how people respond to different incidents of racism has not been the focus (although Lamont et al., 2016 consider this by comparing different groups, but not through in-group comparisons). Similarly, while extensive research has documented and discussed a wide range of responses (see, e.g., Witte, 2018; Drouhot, 2023; Imoagene, 2019), the question of how different understandings of racism and understandings of incidents of racism affect individuals’ responses has remained largely unexplored. The present study is a step towards filling this gap.

In addition, a comparative focus on participants’ understandings and responses allows for differences within groups to be highlighted and opens up a way of explaining them. Such in-group differences in experiences of and responses to exclusion, and especially the question of how to explain them, have increasingly attracted the interest of scholars in ethnic and migration studies. For example, Drouhot (2023), with regard to a group of elite immigrants of North and Sub-Saharan African origin in France, has shown that religious affiliation can explain variation in experiences of racism. In particular, he found that high-status non-Muslim respondents perceived less racial stigma than Muslim respondents. And Doering and Peker (2022) found variation in how Muslims in Quebec experience and respond to secularist restrictions and explained this finding by factors such as whether (1) individuals personally wear religious clothing or (2) have strong social ties to those who experience restrictions more negatively. The second factor points to the importance of how individuals interpret restrictions and thus relates to this study’s focus on people’s understanding of racism and exclusionary incidents.

In this study, the interviewees were all relatively highly educated, occupied a middle-class position in society (or were about to do so after graduation), and lived in either Frankfurt or Hamburg. Following the literature on the “integration paradox,” which argues that a relatively high level of education and a middle-class position make one more likely to frame certain experiences in terms of discrimination (see Schaeffer and Kas, 2024), one would be able to explain Type 1 interviewees, but less so Type 2.

Adding even further complexity to the discussion of how education and class may matter in terms of perceptions of and responses to racism, Drouhot (2023, p. 1434) found that the highly educated professionals he interviewed in France tended to “perceive only moderate levels of racial stigma in their daily lives” (except for Muslim respondents) and to use cultural elitism as a resource to deflect racism rather than directly confront it. The Type 1 interviewees in the present study do not conform to these findings by Drouhot, as they reported many incidents, tended to frame them as “racist” or “discriminatory” and saw directly addressing and confronting racism as an option. We therefore need to look beyond educational status and class if we are to explain why individuals perceive and respond to racist experiences in different ways. This is all the more so because, as this study shows, there are significant in-group differences. The explanation I would like to propose is to consider the country context in relation to the unequal distribution of knowledge about racism.

Given the silence about racism that dominates the German context compared to, for example, the United States, where research shows that African Americans easily recognise racism (which can be explained by the widespread availability of scripts about how racism works in different situations and the widespread awareness and knowledge of racism, not least because of the Civil Rights Movement; see Lamont et al., 2016), describing one’s experiences in terms of racism is not readily available to racialised people in Germany. As a result, and because societal knowledge about the workings of racism is weakly developed, acquiring such knowledge can be more challenging and requires considerable effort to be willing, motivated and ready to educate oneself through reading books and joining networks such as the ISD. For Type 2 interviewees, this was not a priority in their personal lives, whereas Type 1 interviewees had wider access to knowledge about racism, with some of them even being politically active, such as in organising Black History Month, workshops, etc. As a result, knowledge about racism seems to be more widespread among Type 1 interviewees than among Type 2 interviewees. Indeed, knowledge of racism is the most important factor in explaining the differences between the two types and should therefore be taken into account in future research on in-group differences in how people experience and respond to racism. In addition, and concerning contexts other than Germany, which are comparatively silent about racism (such as, e.g., Poland; see Nowicka, 2024) or, on the contrary, more vigilant about racism (such as, e.g., the United States), it would be interesting to examine how knowledge of racism varies at the level of the individual, and whether this varying knowledge is similarly related (as in this study) to how individuals make sense of and respond to racist incidents, with individuals with extensive knowledge having a range of response options at their disposal (of which confrontation is one), and individuals with less extensive knowledge tending to deflate.

Furthermore, and also with a view to future research, it is important to bear in mind that the two types of interviewees presented here are not exhaustive. In particular, four respondents would not fit into either type. Two of them were very knowledgeable about racism and its ramifications and various expressions, but did not have had many experiences of racism in their present lives themselves, which one of them explained by him consequently following a strategy of avoidance of situations in which he would possibly face racism, and the other interviewee referred to her individual characteristics and the environment in which she was lucky enough to live and work. A third interviewee recounted numerous experiences of exclusion, but framed them in terms of feelings of unease only, although she was also very knowledgeable about racism. However, as she also said, her main behavioural strategy was to ignore problematic incidents and not engage in interpreting them. Finally, a fourth interviewee, who reported quite a few experiences but had a less comprehensive understanding of racism, still categorised some of these experiences as “discrimination,” but did so very cautiously. The implication is that, beyond the two (ideal) types presented here, there are not only more types within the larger group of highly educated Black Germans, and possibly also more ways in which experiences, understandings and responses may be related. More research in Germany but also in other contexts is warranted to explore, understand and explain these relationships, and thus to make further progress in understanding in-group differences in how and why people respond to racism as they do.

Of course, focusing on such differences requires a research design that is sensitive to the diversity of individuals’ understandings in the first place. Methodologically, with regard to interview studies, which by and large dominate the study of responses to racism in ethnic and migration studies, this means going beyond analysing respondents’ responses to the interviewer’s direct question about whether they have ever experienced discrimination and/or racism, and focusing on other parts of the interview where, in particular, respondents who do not subscribe to the idea of being racialised or who are reluctant to frame their experiences in terms of racism may report racist experiences by, for example, indicating only feelings of unease or even by normalising such experiences (see Piwoni, 2024a). Such an approach ties in with innovative approaches to studying research participants’ narrations and alternative expressions of racist experiences (through, for example, drawings, the use of photographic images, or photovoice) through a lens that takes affects and emotions seriously (Nowicka and Wojnicka, 2023; Wojnicka and Nowicka, 2023; see also, e.g., Moreno Figueroa, 2008; Cornell and Kessi, 2017).

Importantly, and regardless of the method we choose to elicit participants’ experiences, their ability to talk about these experiences also depends “on the researchers’ sensibilities and competences, as well as on their political positions and goals” (Wojnicka and Nowicka, 2023, p. 2). In the study presented here, I turned the subject of the interview towards racism by asking them at some point (and in cases where they had not previously raised the issue themselves) whether they had had any experiences of exclusion, such as being discriminated against, and only in cases where the interviewees themselves had previously used the term “racism” did I include it in my wording of the question. This is because, as Wojnicka and Nowicka (2023) point out, we as researchers are influenced by culture, and growing up in a culture where “racism” is a word that is used cautiously certainly contributed to my reluctance to use it for one of my central questions. However, I later realised that this reluctance had an advantage in that it gave the interviewees a certain freedom to decide themselves whether to categorise their own experiences as “racist.” On the other hand, of course, the fact that I, the interviewer, had not introduced the word, may have influenced interviewees not to use the word themselves because of social desirability tendencies, which are “more common in research on issues that participants find sensitive or controversial” (Bergen and Labonté, 2020, p. 783). Relatedly, it may be that interviewees avoided such framing because they feared that I, a White researcher, would find it inappropriate, possibly also because they had negative experiences with White people not acknowledging that their experiences were indeed experiences of racism. Notably, some interviewees mentioned having made such experiences. Moreover, and thereby building on Best (2003), the interviews inevitably constituted an interactional context, in which both my own and the interviewees’ racial identities were mobilised. Relatedly, and thereby building further on Best’s (2003) argument that context matters for how racial identities are actively managed and negotiated, it is possible that the interviewees would have been more inclined to speak of “racism” and label certain experiences as “racist” if the interviews had been conducted in English, as the German word “Rassismus” was rarely used in German public discourse prior to the Black Lives Matter protests in Germany in the summer of 2020 (see Zajak et al., 2023).^11^ Despite these possible limitations, this research clearly points to the importance of knowledge about racism and the impact of this knowledge on interviewees’ anti-racist strategies and responses. Overall, when using qualitative interviewing as a method to uncover how respondents experience and respond to racism, it is crucial not to impose our assumptions, but to seek to create and co-create with respondents a space in which their own (affective) understandings of racist experiences, as well as their knowledge about racism can come to the fore. In a context such as Germany, this may mean that interviews about racism may have to do without questions about racism.

## Data Availability

The datasets presented in this article are not readily available because interviewees were guaranteed confidentiality and anonymity. The interview transcripts contain information that may identify participants. Requests to access the datasets should be directed to eunike.piwoni@uni-passau.de.

## Footnotes

^1^Although often mentioned together, sometimes used interchangeably and arguably related, the four terms have distinct meanings (see, e.g., Pager and Shepherd, 2008 for racial discrimination; Link and Phelan, 2001 for stigmatisation; Essed, 1991 for racism and Lamont et al., 2016 for ethnoracial exclusion). While discrimination is often defined as the unequal treatment of individuals or groups on the basis of, e.g., ethnicity or race, stigmatisation describes processes by which individuals or groups are devalued on the basis of marks or attributes regarded as discrediting. Racism can be understood, as Essed (1991, pp. 39, 42) suggests, “in terms of cognitions, actions, and procedures that contribute to the development and perpetuation of a system in which Whites dominate Blacks,” with ideology playing a “structuring role.” Ethnoracial exclusion has not been theorised in its own right, but has been used to describe exclusion based on race, ethnicity, or national origin, and primarily in contexts, where the existence of racism is highly contested (see Lamont et al., 2016, p. 313, Note 15). For the present research, which focuses on how individuals respond to (experiences of) racism, the following concepts and definitions from Lamont et al. (2016) have been used: “experiences of discrimination” and “experiences of stigmatization,” both of which fall under the broader terms of “experiences of ethnoracial exclusion” or “experiences of racism” respectively. Definitions of the terms are provided in the main text when reviewing the literature in the field, and in the data and methods sections.

^2^“Migrant background” here refers to the fact that the interviewees had at least one parent who did not acquire German citizenship at birth. This may be reminiscent of the official category “persons with a migration background” [Personen mit Migrationshintergrund] used in the German microcensus and in reporting by the Federal Office for Migration and Refugees, which is operationalised as persons who themselves or at least one of their parents did not acquire German citizenship with birth (see Will, 2019). In official reporting, the term is now increasingly being replaced by the category “persons with a migration history” [Personen mit Einwanderungsgeschichte], which is operationalised as immigrants and their direct descendants. “Persons with a migration background” is also used in public discourse in Germany and has been criticised for “othering” because, as Will (2019, p. 535) argues, “inherited citizenship and ancestry is in the center of the definition” (see also Moffitt and Juang, 2019, p. 657 who argue that the use of “German” without qualification is “common parlance for *white* German” and “used to reference individuals from those nations *perceived* as not having heritage from elsewhere”).

^3^This research is situated in the sociological literature, and more specifically in the study of migration, race and ethnicity. While sociological research has tended to highlight differences between groups in how they cope with ethnoracial exclusion, and has only recently begun to consider in-group differences (a research gap that this article aims to fill), psychologists have pointed to variability in individuals’ responses based on concepts such as resilience. Leanne Son Hing (2012), for example, has outlined the role of primary appraisals (i.e., how harmful and self-relevant individuals perceive an incident of stigmatisation to be) and secondary appraisals (i.e., whether or not they believe they have the resources to cope) in how individuals respond to stigmatisation. In addition, Son Hing has pointed to moderators that influence these appraisals, such as group identification or one’s assessment of one’s own resources. However, the argument of this article is less concerned with individual stress reactions and how to explain resilience at the individual level. Instead, it looks at in-group differences in how individuals respond to racism, foregrounding the idea that these differences are related to how an individual understands a racist event and racism more generally. As Essed (1991) and Piwoni (2024a) argue, access to knowledge about racism varies between societies and also between different groups in society. It is also, as this article shows, unevenly distributed within particular groups.

^4^Importantly, this does not mean that racism is located in people’s emotions or cannot (per se) be understood rationally. Instead, Bonilla-Silva’s (2019, p. 2) point is that racism is not limited to “presumably objective practices and mechanisms” and that “racialized emotions,” which he defines as emotions that are related to race and that people experience in, e.g., interracial interactions, are “fundamental social forces shaping the house of racism.” At the level of the individual, this means that, even when actors do not consciously engage with racism, they “feel the emotional weight of their categorical location.”

^5^Some interviews were conducted before the outbreak of COVID-19, some during a lockdown (which basically resulted in interviewees offering more time slots for interviews), and some at non-lockdown times. In terms of content, I found that two interviewees I spoke to after the COVID-19 outbreak would report racist incidents related to wearing a mask, but otherwise, and particularly in terms of the main argument of the paper, the pandemic did not feature prominently in interviewees’ narratives of experiences of exclusion. Furthermore, 11 interviews were conducted before the Black Lives Matter (BLM) protest wave in Germany in summer 2020, while 10 interviews were conducted in autumn 2020 and winter 2020/21. While these protests were mentioned in seven of these 10 interviews, the interviewees did not elaborate on them (which were already a past event at the time of the interview), and some were convinced that such protests did not have any long-term effects in terms of actually fighting racism, while one interviewee said that he was sceptical about BLM as a protest movement originating in the US with its different history of racism. Certainly, the BLM movement has been significant in terms of driving a mass-mediated debate about racism in Germany (see Zajak et al., 2023), and research has shown that macro-level events do indeed have an impact on how individuals make meaning (see, e.g., Moffitt et al., 2024). However, this study was not designed to detect such an effect; moreover, the group of participants I interviewed after the wave of protests also included interviewees who tended to normalise exclusionary experiences and not to label them as “racist” (see Findings section), and there were no respondents who described BLM as an event that changed their views or perceptions.

^6^One respondent said she was in her late 20s and was not included in the calculation.

^7^Afro-Diasporic Academic Network; https://ada-netzwerk.com/.

^8^Notably, this definition of “experiences of stigmatization” includes experiences of being stereotyped, but also experiences of microaggressions, which have been defined as “brief and commonplace daily verbal, behavioral, or environmental indignities, whether intentional or unintentional, that communicate hostile, derogatory, or negative racial slights and insults toward people of color” (Sue et al., 2007, p. 273).

^9^Notably, there were also Type 1 interviewees who had been taught about racism by their parents. Research has pointed to the impact of racism-conscious socialisation and parenting, through, for example, proactive or reactive preparation for bias (see, e.g., Iqbal, 2014; see also Rogers et al., 2024).

^10^See https://isdonline.de/ueber-uns/.

^11^However, about half of all interviews were conducted before these protests, and the other half in autumn 2020 and winter 2020/21, and yet both types of interviewees were present in all interviews, regardless of when they were conducted.
